# Effects of Auraptene on IGF-1 Stimulated Cell Cycle Progression in the Human Breast Cancer Cell Line, MCF-7

**DOI:** 10.1155/2012/502092

**Published:** 2012-12-18

**Authors:** Prasad Krishnan, Heather Kleiner-Hancock

**Affiliations:** ^1^Department of Pharmacology, Toxicology & Neuroscience, Louisiana State University, Health Sciences Center, 1501 Kings Highway, Shreveport, LA 71130, USA; ^2^Department of Veterinary and Biomedical Sciences, Center for Molecular Toxicology and Carcinogenesis, Pennsylvania State University, University Park, PA 16802, USA

## Abstract

Auraptene is being investigated for its chemopreventive effects in many models of cancer including skin, colon, prostate, and breast. Many mechanisms of action including anti-inflammatory, antiproliferative, and antiapoptotic effects are being suggested for the chemopreventive properties of auraptene. We have previously shown in the *N*-methylnitrosourea induced mammary carcinogenesis model that dietary auraptene (500 ppm) significantly delayed tumor latency. The delay in time to tumor corresponded with a significant reduction in cyclin D1 protein expression in the tumors. Since cyclin D1 is a major regulator of cell cycle, we further studied the effects of auraptene on cell cycle and the genes related to cell cycle in MCF-7 cells. Here we show that auraptene significantly inhibited IGF-1 stimulated S phase of cell cycle in MCF-7 cells and significantly changed the transcription of many genes involved in cell cycle.

## 1. Introduction

Breast cancer has been one of the most common cancers among women in the United States. The estimates for 2012 are 226,870 new cases of breast cancer and 39,510 deaths due to breast cancer in women in the United States [1]. In addition to the incidence and death in women, 2190 new cases of breast cancer incidence and 410 deaths are expected in men in 2012 [1]. The current treatment modalities have severe adverse effects including endometrial cancer, cardiotoxicity, leukemia, blood clots, nausea, and vomiting, [1]. Hence, chemoprevention of cancer could be a better strategy.

The idea of cancer chemoprevention has received more attention since the promotion stage of breast cancer generally is a lengthy process that could be reversible [2]. Many natural products are under study to prevent cancer including breast cancer. One among those is auraptene from citrus fruits [3]. Previous studies with auraptene showed its chemopreventive effects in rodent models of many cancers including colon [4–7], esophagus [8], tongue [9], liver [10, 11], and skin [3]. Our study demonstrated the chemopreventive effects of auraptene against breast cancer [12]. These studies in human breast carcinoma cell lines, MDA-MB-231, and MCF-7 indicated the anti-proliferative effects of citrus auraptene. Subsequently an *in vivo* study demonstrated that dietary auraptene (500 ppm in the diet) decreased mammary carcinoma incidence and delayed median time to tumor in *N*-methylnitrosourea (MNU) treated rats. HPLC analysis of the mammary tissues from auraptene treated rats detected auraptene in the low *μ*M concentrations at both 200 and 500 ppm dose [12].

**Figure 1:**
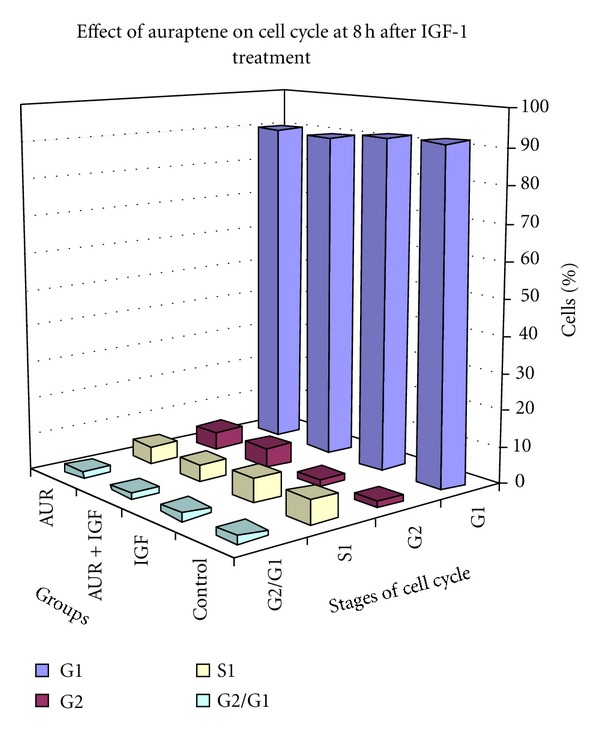
Cell cycle analysis of MCF-7 cells in the presence of IGF-1 at 8 h. MCF-7 cells were serum starved for 24 h. At 22 h after serum starvation the cells were treated with 10 *μ*M auraptene in 0.01% DMSO. At 24 h serum starvation, the cells were treated with IGF-1 (10 ng/mL). After 8 h of IGF-1 treatment, the cells were harvested and processed for cell cycle analysis by flow cytometry.

Many mechanisms have been attributed to the chemopreventive effects of auraptene. They include inhibition of polyamine synthesis [9, 13], induction of detoxifying enzymes [14, 15], induction of apoptosis [3, 16], inhibition of metalloproteinase [17, 18], and inhibition of cholesterol esterification [19] to name a few. Recently, Medina and colleagues reported that auraptene modulated genes under the transcriptional control of estrogen [19]. In our *in vivo* study, further analysis of the mammary tissues from rats showed that cyclin D1 protein was significantly reduced in auraptene 500 ppm treated animals [12]. Cyclin D1 is a key regulatory protein in cell cycle (Figure 1). The D-type cyclins control the G1 to S transition during the cell cycle along with E-type cyclins [20]. In up to 50% of primary breast cancers the overexpression of cyclin D1 mRNA and protein has been observed [20]. Thus cyclin D1 is one of the most overexpressed oncogenes in human breast cancer. Cyclin D1 overexpression is predominantly found in estrogen receptor positive breast cancer, which is a major subtype of human breast cancer [20]. 

Since cyclin D1 protein expression was reduced in mammary tissues of auraptene treated rats, we explored the effect of auraptene on cell cycle in human breast carcinoma cell line MCF-7, which is positive for the estrogen receptor. The results shed more light on the inhibitory effects of auraptene on cell cycle machinery.

## 2. Materials and Methods

### 2.1. Chemicals

Auraptene was purchased from LKT Laboratories Inc. (St. Paul, MN). Recombinant human IGF-1 was obtained from Peprotech Inc. (Rocky Hill, NJ).

### 2.2. Cell Culture

Human MCF-7 mammary adenocarcinoma cells were purchased from ATCC (Manassas,VA). The cells were grown in sterile humidified chamber maintained at 37°C and 5% CO_2_: 95% air. The MCF-7 cells were grown and propagated in complete DMEM medium.

### 2.3. Cell Cycle Analysis

The effect of auraptene on cell cycle was studied in MCF-7 cells. Briefly, 1 × 10^6^ cells were plated in eight 10 cm dishes, with duplicate dishes in each group. There were four groups: control (DMSO), IGF-1 only, IGF-1 + auraptene, and auraptene only. The cells were serum starved on the next day of plating. After 22 h of serum starvation, the IGF-1 + auraptene and auraptene only groups were pretreated with 10 *μ*M of auraptene in DMSO (0.1% v/v). The control and IGF-1 only groups received vehicle. At 24 h of serum starvation, the IGF-1 only and IGF-1 + auraptene groups received 10 ng/mL of IGF-1. After 8 h and 24 h of IGF-1 treatment, the cells were harvested and centrifuged and the supernatant was removed. The pellets were then washed twice with ice cold PBS. The cells were counted and then fixed with ice cold 70% ethanol and kept at 4° overnight. The next day, the cells were centrifuged, the ethanol was removed and the cells were washed twice with ice cold PBS. Then, to each tube 500 *μ*L of the propidium iodide solution mix was added (propidium iodide solution mix was prepared by adding 10 *μ*L of Triton-X 100 (10% stock), 200 *μ*L of 10 mg/mL RNAse solution, and 400 *μ*L of 0.5 mg/mL propidium iodide solution to 10 mL PBS) and incubated for 30 minutes at room temperature, followed by cell cycle analysis using flow cytometry. The experiment was repeated 3 times. The flow cytometry was conducted using a BD LSRII (BD Biosciences) in the Research Core Facility at the LSUHSC-Shreveport. The instrument has a Coherent Sapphire laser for 488 nm excitation, a JDS Uniphase HeNe laser for 633 nm excitation, as well as a Coherent VioFlame for 450 nm excitation. The data were analyzed with FACS Diva (BD Biosciences) and ModFit LT software (Verify Software House).

### 2.4. qRT-PCR

A qRT-PCR array was done to analyze changes in gene expression of cell cycle related genes by auraptene. For this experiment, 1 × 10^5^ cells were plated in eight 6 cm dishes, with duplicate dishes in each group. There were four groups: control, IGF-1 only, IGF-1 + auraptene, and auraptene only. The cells were serum starved on the next day of plating. At 22 h of serum starvation, the IGF-1 + auraptene and auraptene only groups were pretreated with 10 *μ*M of auraptene in DMSO (0.1%, v/v). The control and IGF-1 only groups received vehicle. At 24 h of serum starvation, the IGF-1 only and IGF-1 + auraptene groups received 10 ng/mL of IGF-1. After 8 h and 24 h of IGF-1 treatment, the cells were processed for RNA isolation as per the manufacturer's instructions (Qiagen, RNeasy minikit). The experiments were conducted in triplicate. The isolated RNA was treated with DNAse to remove any genomic contamination as per the manufacturer's suggested protocol (Promega). The DNA free RNA was then analyzed for quality on Agilent Analyzer. After confirming the quality of RNA, 1 *μ*g of RNA was converted to cDNA as per the directions from the manufacturer (Quanta Biosciences). The 1 : 10 dilution of cDNA was used for amplification with Perfecta SYBR Green Fast Mix for iQ (Quanta Biosciences) in the PCR Array (Human Cell Cycle Tox and Cancer 96 StellARray qPCR array, Lonza). The results from all experiments were analyzed using the Global Pattern Recognition Software from Bar Harbor BioTechnology, available on Lonza's website (http://array.lonza.com/stellarrays/). The experiment was repeated 3 times at 8 h and also at 24 h after IGF treatment. GPR fold changes were considered significant at *P* ≤ 0.05.

### 2.5. Statistical Analysis

The analysis of cell cycle data was done by One-Way ANOVA followed by Tukey/Kramer post hoc test, *P* < 0.01. The results from qRT-PCR array studies were analyzed with the Global Pattern Recognition Software available on Lonza's website (http://array.lonza.com/stellarrays/), *P* < 0.05.

## 3. Results 

### 3.1. No Significant Change in the Percentage of Cells in the S Phase after 8 h of IGF-1 Treatment

After 8 h of IGF-1 treatment, the harvested cells were run by flow cytometer to analyze the percentage of cells in the different phases of cell cycle (Figure 1 and Table 1). Most of the cells of the control group were in the G1 phase (92%), and there were no significant differences in the percentages of cells in the G1 phase among the treatment groups. IGF-1 did not produce any significant increase in the percentage of cells in S phase of the cell cycle at 8 h and no significant reduction in S phase was found in the auraptene treated cells. The percentage of cells in G2 in the control and the treatment groups also were almost the same. The effects of auraptene on cell cycle in the absence of IGF-1 was no different than the control group at 8 h. Also, there were no apparent differences in the ratio of G2/G1.

### 3.2. Auraptene Significantly Reduced the Percentage of Cells in the S Phase after 24 h of IGF-1 Treatment

**Figure 2:**
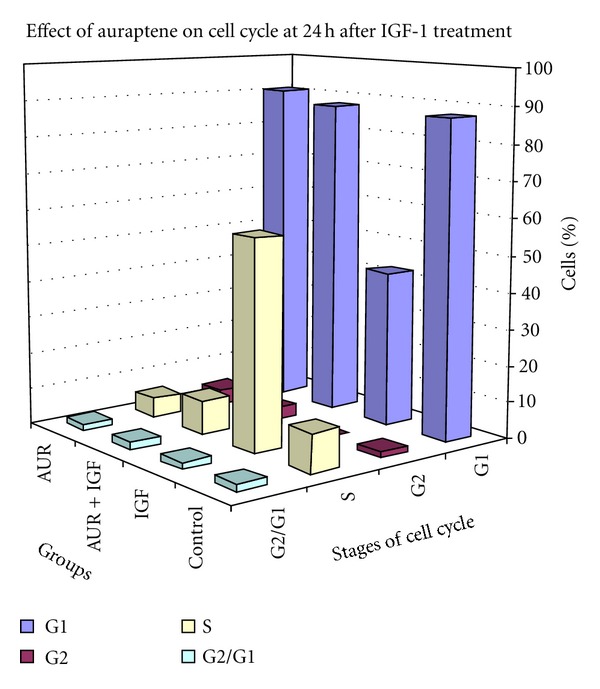
Cell cycle analysis of MCF-7 cells in the presence of IGF-1 at 24 h. MCF-7 cells were serum starved for 24 h. At 22 h after serum starvation the cells were treated with 10 *μ*M auraptene in 0.01% DMSO. After 24 h of serum starvation, the cells were treated with IGF-1 (10 ng/mL). After 24 h of IGF-1 treatment, the cells were harvested and processed for cell cycle analysis by flow cytometry.

After 24 h of IGF-1 treatment, the harvested cells were run by flow cytometer to analyze the percentage of cells in the various phases of cell cycle. IGF-1 treatment resulted in a significantly decreased percentage of cells in the G1 phase compared to all the other groups from 87% in the control to 46% in the IGF-1 treated group (Figure 2 and Table 2). There was a corresponding increase in the percentage of cells in S phase in the IGF-1 treated group (from 10% in the control group to 57% in the IGF-1 treated group). Auraptene pretreatment significantly reduced the percentage of cells in S phase in the IGF-1 treated cells and appeared to restore the cells back to control levels of G1. The effects of auraptene on cell cycle in the absence of IGF-1 were no different than the control group at 24 h. Also, there were no apparent differences in the ratio of G2/G1.

### 3.3. Auraptene Pretreatment in IGF-1 Treated MCF-7 Cells Significantly Modulated Several Genes Involved in Cell Cycle Regulation, Compared to IGF-1 Alone Treated Cells after 8 h of IGF-1 Treatment

In Table 3, the significant changes in the gene transcript level with auraptene pretreatment in IGF-1 treated cells when compared to the IGF-1 alone treated cells at 8 h time point are shown. There were significant changes in 9 genes, with 6 downregulated and 3 upregulated ones. The downregulated genes were *E2F1* (E2F transcription factor 1), *CDC45L* (cell division cycle 45 homolog), *E2F2* (E2F transcription factor 2), *MCM3* (minichromosome maintenance complex component 3), *MCM6* (minichromosome maintenance complex component 6), and *UHRF1* (ubiquitin-like with PHD and ring finger domains 1). The upregulated genes include *CDKN2B* (cyclin-dependent kinase inhibitor 2B), *DDIT3* (DNA-damage-inducible transcript 3), and *JUN *(jun oncogene). The IGF-1 alone treatment resulted in the increased transcription of only 2 genes, *MCM6 *and *ORC1L *(origin recognition complex, subunit 1-like), which are key regulators of cell replication complex.

### 3.4. Auraptene Pretreatment in IGF-1 Treated MCF-7 Cells Significantly Modulated Several Genes Involved in Cell Cycle Regulation, Compared to IGF-1 Alone Treated Cells after 24 h of IGF-1 Treatment

In Table 4, the significant changes in the gene transcript level with auraptene pretreatment in IGF-1 treated cells when compared to the IGF-1 alone treated cells at 24 h time point are shown. There were 14 genes that changed significantly. Ten genes were downregulated while 4 were upregulated. The downregulated genes were *CDC45L* (cell division cycle 45 homolog), *CDC2* (cyclin-dependent kinase 1), *CCNA2* (cyclin A2), *KIF20B *(kinesin family member 20B), *CHEK1* (CHK1 checkpoint homolog), *CDKN2C* (cyclin-dependent kinase inhibitor 2C), *CHEK2* (CHK2 checkpoint homolog), *E2F1* (E2F transcription factor 1), *UHRF1* (ubiquitin-like with PHD and ring finger domains 1), and *CCNB2* (cyclin B2). The upregulated genes were *DDIT3* (DNA-damage-inducible transcript 3), *CDKN2B *(cyclin-dependent kinase inhibitor 2B), *GADD45A* (growth arrest and DNA-damage-inducible, alpha), and* DUSP1* (dual specificity phosphatase 1). E2F1, CDC45L, UHRF1, DDIT3, and CDKN2B were modulated at both 8 h and 24 h.

## 4. Discussion

Here, the effects of auraptene on cell cycle and genes involved in the mammalian cell cycle have been described. This study was conducted after finding the inhibitory effect of auraptene on cyclin D1 protein expression in MCF-7 cells and rat mammary tumors. We previously showed that in MCF-7 cells 10 *μ*M auraptene reduced the cyclin D1 protein expression by about 40% after treating with IGF-1. In the subsequent animal study, auraptene at 500 ppm dose in the diet significantly delayed the time to tumor compared to the MNU only group. The rat mammary tumors from auraptene 500 ppm group showed significant reduction in cyclin D1 protein expression [12]. Since cyclin D1 is a key protein that regulates G1/S transition in cell cycle [21], we hypothesized that auraptene will inhibit the progression of cell cycle. To our knowledge this is the first study to show a detailing of the effect of auraptene on IGF-1 induced stimulation of the cell cycle in a breast cancer cell line. Auraptene significantly reduced the percentage of cells in the S phase at the 24 h time point. This effect was not observed at the 8 h time point, even though auraptene reduced cyclin D1 expression after 8 h in MCF-7 cells [12]. This might be due to the fact that inhibition of cyclin D1 is an earlier event, which later resulted in slowing down the progression of cell cycle. The mitogenic activity of IGF-1 was not seen at 8 h, since there was no significant increase in the percentage of cells in the S phase compared to the control cells. At 24 h, a significant increase in the percentage of cells in the S phase was observed. Correspondingly, there was a significant reduction in the percentage of cells in the G1 phase of IGF-1 only group. IGF-1 was used to stimulate the serum starved MCF-7 cells because IGF-1 has been shown to be in high concentration in rats treated with MNU [22], which is the same carcinogen we used in our rat study with auraptene [12]. Elevated levels of IGF-1 are also found in human breast cancer patients [23]. Obese women, who are at higher risk to get breast cancer postmenopausally, also have higher IGF-1 blood levels [24]. It might be interesting to see the effect of auraptene in presence of other mitogens. 

In the current study, after auraptene pretreatment, we saw a time dependent change in the transcriptional machinery associated with cell cycle along with the inhibition of S phase. At 8 h time point, significant changes were observed in 9 genes and at 24 h in 14 genes. Among these genes, 4 genes had a greater fold of change at the latter time point. Therefore, from the fact that these genes are still transcriptionally reduced, it is concluded that the effect of auraptene on cell cycle could persist for at least 24 h. This could be the effect of parent compound or its active metabolites. The major metabolites of auraptene are umbelliferone and 7-ethoxycoumarin which also have been shown to possess chemopreventive effects [25].

The cyclin D1 gene was also one of the cell cycle genes whose changes were analyzed along with the other mentioned genes. We found that there was no significant change in the mRNA level of cyclin D1 in the cells treated with IGF-1 and auraptene (data not shown). Therefore, auraptene's effect on cyclin D1 could be posttranscriptional. Previous studies have shown that auraptene changed the protein levels of COX-2, iNOS, and pro-MMP-7 without changing their transcript levels [18, 26]. In the human colorectal adenocarcinoma cell line HT-29, auraptene disrupted the translation of proMMP-7 protein synthesis by decreasing phosphorylation levels of 4E binding protein (4EBP)1 and eukaryotic translation initiation factor (eIF)4B [18]. These data along with our results point to the fact that auraptene might be acting on the translational machinery of the cells. 

Many genes that were modulated by auraptene have been shown to be relevant in many cancers. Most of the prostate and breast-cancer cell lines studied had reduced copy number of *CDKN2B *gene [27], whereas in this study we found it upregulated with time after auraptene pretreatment. *CDKN2B, *an inhibitor of cyclin D1 induced cell cycle progression from G1 phase to S phase, could contribute to the auraptene induced inhibition of cell cycle at G1 phase. *CDKN2B* was found to be increased by auraptene to almost similar extent at both 8 h and 24 h whereas the S phase inhibition was evident at 24 h. This might be because regulation of *CDKN2B *might be an earlier event that leads to further inhibition of cell cycle machinery at a later time point. The E2F freed from pRB by the cyclin D-CDK complex upregulates the transcription of genes involved in DNA-replication—*MCM components*, *cdc6,* and *cyclin E* [28]. The *E2F* genes are deregulated in many cancers by various mechanisms like *overexpression* of cyclin D1, loss of pRB, and expression of human papillomavirus (HPV) oncoprotein E7, to name a few [29]. CDC45 protein has been shown to be overexpressed in various human cancer cell lines, including MCF-7 [30]. UHRF1 is under the transcriptional control of E2F1 transcription factor [31]. UHRF1 has been shown to be upregulated in cancer of breast, prostate, pancreas, and so forth [31–33]. Thus at the earlier time point in this study, the downregulation of cyclin D1 could have resulted in the reduced transcription of E2Fs and their downstream targets like UHRF1 and MCM complexes resulting in the initiation of cell cycle arrest. JUN or c-Jun is a component of the composite transcription factor activating protein-1 (AP-1) and promotes cell proliferation [34, 35]. C-Jun is overexpressed in many human cancers and contributes to the invasiveness of human breast cancer cells [36]. In this study, we found *JUN *upregulated at 8 h time point only and not at 24 h time point that corresponds with the inhibition of S phase at 24 h by auraptene. 

At the later time point auraptene pretreatment resulted in greater change in the gene expression of CDC45L, E2F1, UHRF1, DDIT3, and CDK2NB. The other genes modulated by auraptene also have been shown to be deregulated in many cancers. The DUSP1 transcript was dramatically decreased in colorectal cancer compared to normal cells [37, 38]. Li and colleagues [38] showed that DUSP1 (MKP1) is a transcriptional target of p53 that inhibits MAPK pathway activation of cell cycle progression. In estrogen dependent breast cancer cells, estrogen was shown to increase the expression of cyclin B2 protein and promote cell proliferation [39]. CHEK1 *overexpression* has been seen in human colorectal cancer correlated with advanced tumor and poor prognosis [40].* CDKN2C *is the gene that codes for the cyclin dependent kinase 4 inhibitor C or p18. Mutation in *p18 *has been found in some human breast tumors [41]. Similar to *CDKN2B*, *CDKN2C* also inhibits cyclin induced G1/S transition. Therefore the increased expression of *CDKN2C *might also be playing an important role in auraptene induced inhibition of cell cycle at G1 phase. However these 2 genes, having similar functions, are modulated differently by auraptene at 8 h and 24 h. KIF20B is a member of the kinesin-6 family [42]. It is involved in cytokinesis [43]. Several members of the KIF family are upregulated in cancer. In lung cancer, the KIF4A gene was observed to be highly transactivated [44]. In the majority of glioma cell lines, 3 genes were *overexpressed*:* KIF1C, KIF3C, *and *KIF21B *[45]. KIF20B was found to be highly expressed in a majority of human invasive bladder cancers [46]. Thus the overall gene changes brought about by auraptene pretreatment inhibited the progression of cell cycle at the G1 phase in MCF-7 cells. 

A few of the above-mentioned genes have been identified as targets of anticancer therapy. CDC2 is also known as CDK1. CDK modulators have been investigated in clinical trials against many cancers. Flavopiridol, a CDK1 and CDK2 inhibitor [47], is being clinically tested against gastric cancer, leukemia, and head and neck cancer [48]. Another CDK1 modulator currently in clinical trials is UCN-01 [49]. In a cell cycle gene array experiment in MCF-10F cells with etodolac, a COX-2 inhibitor, *CCNA2 *was one of the prominently altered genes [50]. In PC-3 prostate cancer cells, luteolin reduced the transcription of *CCNA2 *along with that of other cell cycle genes and inhibited the proliferation of those cells [51]. Since auraptene modulated these genes, further analysis of its potential as a dietary chemopreventive agent needs to be carried out.

## 5. Conclusion

Here we investigated the role of auraptene on cell cycle progression of human breast carcinoma cell line, MCF-7, and we showed the inhibitory effect of auraptene on cell cycle in MCF-7. Auraptene significantly reduced the percentage of cells undergoing S phase after 24 h of IGF-1 treatment. There were several genes involved in cell cycle that were significantly modulated with auraptene pretreatment. More genes were modulated at 24 h time point and correspondingly auraptene pretreatment showed inhibition of the cell cycle progression. Overall, auraptene pretreatment produced significant increase in the mRNA level of genes known to be upregulated prior to cell cycle arrest and apoptosis. There was a significant decrease in the mRNA level of genes that promote G1/S transition and DNA replication. Further studies on the effect of auraptene on cell cycle progression and protein translational machinery in different breast cancer cell lines will provide us with more information about its chemopreventive properties. However, the results obtained with auraptene in MCF-7 cells throw more light on cell cycle inhibition as one of its chemopreventive mechanisms. Since breast cancer is a multifactorial disease, a combination of drugs should be tried for chemoprevention studies and auraptene could be one of the candidates.

## Figures and Tables

**Table 1:** Average percentage of cells at 8 h time point.

Stage of cell cycle	Control	IGF	IGF + auraptene	Auraptene
G1	91.8 ± 0.5	91.3 ± 0.2	89.8 ± 0.6	90.2 ± 0.94
G2	1.4 ± 0.7	1.9 ± 0.9	5.3 ± 0.5	4.8 ± 0.8
S	6.8 ± 0.9	6.7 ± 1.2	4.8 ± 0.2	4.9 ± 0.3
G2/G1	2.0 ± 0.1	1.9 ± 0.1	1.8 ± 0.0	1.9 ± 0.0

Figures represent means ± SEM (*n* = 3).

**Table 2:** Average percentage of cells at 24 h time point.

Stage of cell cycle	Control	IGF	IGF + auraptene	Auraptene
G1	87.4 ± 1.9	42.6 ± 0.4^a^	87.4 ± 2.0	90.6 ± 1.0
G2	1.9 ± 1.5	0.1 ± 0.1	3.2 ± 1.1	4.1 ± 0.4
S	10.7 ± 0.5	57.2 ± 3.4^a^	9.5 ± 0.9	5.4 ± 0.7
G2/G1	2.0 ±0.0	2.0 ± 0.0	2.0 ± 0.0	1.9 ± 0.0

Figures represent means ± SEM (*n* = 3). ^a^Significantly different from control *P* ≤ 0.01.

**Table 3:** Significant GPR fold change in the mRNA of target genes related to cell cycle in IGF-1 + auraptene treated cells compared to cells treated with IGF-1 alone at 8 h.

Gene	Function	GPR fold change
E2F1	Act in the G1/S transition	−7.88
CDC45L	DNA replication	−6.51
E2F2	Act in the G1/S transition	−9.81
MCM3	Integral part of the prereplication complex during cell cycle	−6.08
MCM6	Integral part of the prereplication complex during cell cycle	−3.71
UHRF1	Recruits histone deacetylase during cell cycle. Major role in G1/S transition	−17.01
CDKN2B	Prevent activation of CDK by cyclin D1	5.83
DDIT3	Arresting cell cycle after DNA damage	9.36
JUN	Form AP-1 and promote cell proliferation	3.65

**Table 4:** Significant GPR fold change in the mRNA of target genes related to cell cycle in IGF-1 + auraptene treated cells compared to cells treated with IGF-1 alone at 24 h (the genes significantly changed at both 8 h and 24 h time points are shown in bold).

Gene	Function	GPR fold change
**CDC45L**	**DNA replication**	**−20.71**
CDC2	Promotes G1/S transition	−38.29
CCNA2	Promotes cell cycle in the mitosis phase	−20.25
KIF20B	Involved in the cytokinesis, the final phase of cell division	−32.48
CHEK1	Inhibits cell division	−9.01
CDKN2C	Inhibits G1/S entry	−8.98
CHEK2	Cell cycle arrest. Activated in response to DNA damage.	−10.83
**E2F1**	**Act in the G1/S transition**	**−10.44**
CCNB2	Promotes cell division, active in G2/M phase	−6.81
**UHRF1**	**Recruits histone deacetylase during cell cycle. Major role in G1/S transition**	**−45.85**
**DDIT3**	**Arresting cell cycle after DNA damage**	**53.22**
**CDKN2B**	**Prevent activation of CDK by cyclin D1**	**6.14**
GADD45A	Participates in arresting cell cycle after DNA damage	10.16
DUSP1	Inactivates MAPK phosphorylation and inhibits proliferation	7.51
